# Endothelial Progenitor Cells Affect the Growth and Apoptosis of Renal Cells by Secreting Microvesicles Carrying Dysregulated miR-205 and miR-206

**DOI:** 10.1155/2023/4397829

**Published:** 2023-02-16

**Authors:** Yunbo Zhang, Yanling Shen, Yanling Song, Yunyun Lin, Ying Wang, Minghui Chen, Huade Mai, Shenhong Gu

**Affiliations:** ^1^Department of General Practice, The First Affiliated Hospital of Hainan Medical University, Hainan 570102, China; ^2^Hainan Medical University, Hainan 570000, China; ^3^Key Laboratory of Tropical Cardiovascular Diseases Research of Hainan Province, Hainan 570102, China

## Abstract

**Background:**

This study investigated the mechanism of microRNA (miRNA, miR) in microvesicles (MVs) secreted by endothelial progenitor cells (EPCs) involved in renal function in vivo and in vitro injury repair of rat primary kidney cells (PRKs).

**Methods:**

Gene Expression Omnibus analysis of potential target miRNAs in nephrotic rats. Real-time quantitative polymerase chain reaction verified the correlation of these miRNAs and screened the effective target miRNAs and their downstream putative target mRNAs. Western blot analyzes the protein levels of DEAD-box helicase 5 (DDX5) and the activation of the proapoptotic factor caspase-3/9 (cleaved). Dil-Ac-LDL staining, immunofluorescence, and a transmission electron microscope (TEM) were used to identify the successful isolation of EPCs and PRKs and the morphology of MVs. Cell Counting Kit-8 was used to detect the effect of miRNA-mRNA on the proliferation of PRKs. Standard biochemical kits were used to detect biochemical indicators in rat blood and urine. Dual-luciferase analysis of miRNA binding to mRNA was conducted. The effect of miRNA-mRNA interaction on the apoptosis level of PRKs was analyzed by flow cytometry.

**Results:**

A total of 13 rat-derived miRNAs were potential therapeutic targets, and miR-205 and miR-206 were screened as the targets of this study. We found that the EPC-MVs alleviated the increase of blood urea nitrogen and urinary albumin excretion and the decrease in creatinine clearance caused by hypertensive nephropathy in vivo. The effect of MVs in improving renal function indicators was promoted by miR-205 and miR-206 and inhibited by knockdown of expressed miR-205 and miR-206. In vitro, angiotensin II (Ang II) promoted growth inhibition and apoptosis of PRKs, and similarly, dysregulated miR-205 and miR-206 affected the induction of Ang II. We then observed that miR-205 and miR-206 cotargeted the downstream target DDX5 and regulated its transcriptional activity and translational levels, while also reducing the activation of proapoptotic factors caspase-3/9. Overexpressed DDX5 reversed the effects of miR-205 and miR-206.

**Conclusion:**

By upregulating the expression of miR-205 and miR-206 in MVs secreted by EPC, the transcriptional activity of DDX5 and the activation of caspase-3/9 can be inhibited, thereby promoting the growth of PRKs and protecting the injury caused by hypertensive nephropathy.

## 1. Introduction

The kidney is one of the major organs affected by hypertensive target organ damage [1, 2]. Hypertensive nephropathy (HN) is clinically characterized by progressive renal fibrosis and inflammation [3, 4], and prolonged hypertension can eventually lead to renal failure [4]. However, the current treatment of HN is still at the level of eliminating symptoms, and the research on the pathogenesis of HN is still unclear. It is generally believed that urinary albumin excretion (UAE) > 20 mg/24 h in Spontaneous Hypertension Rat (SHR) can be considered HN.

Endothelial progenitor cells (EPCs) are a type of stem cells with angiogenic and tissue repair capabilities [5]. Evidence shows that endothelial progenitor cells can improve renal function in patients with diabetic nephropathy [6], possibly because of the beneficial therapeutic effects of endothelial progenitor cell-derived microvesicles (MVs) in various diseases [7]. Many reports have confirmed that the protective effect of EPCs is closely related to the release of MVs [8]. The MVs secreted by EPCs can be absorbed by cells, thereby reducing damage [9] and repairing tissues [10]. The properties of EPC-MVs are similar to those of EPCs. However, the underlying molecular mechanism of EPC repairing renal injury through MVs is still unclear.

MicroRNAs (miRNA, miR) are small noncoding RNAs involved in the progression and treatment of various diseases including HN [11, 12]. Evidence suggests that MVs can ameliorate renal injury by delivering miRNAs [13, 14]. It is well known that dysregulated miRNAs can affect the occurrence and development of various diseases [15, 16], including HN [17]. miRNAs affect renal function by binding to the 3′-UTR of downstream mRNAs and regulating mRNA transcription and translation [18, 19]. The levels of angiotensin II (Ang II) and its receptors in the kidneys of hypertensive rats are much higher than those of Wistar-Kyoto (WKY) rats [20]. Ding et al. believed that angiotensin II-mediated urinary albumin, blood urea nitrogen (BUN), and creatinine clearance (Scr) can be improved by miR-101a by blocking nuclear factor kappa-B signaling [3]. Downregulated miR-205 was positively correlated with BUN and creatinine (Cr) levels in patients with renal injury [21]. After overexpression, miR-205 attenuated sepsis-induced acute kidney injury [22]. miR-206 binds to DEAD-box helicase 5 (DDX5) to inhibit the activation of NLR family pyrin domain containing 3 inflammasome and alleviate acute kidney injury [23].

These proteases, cysteinyl aspartate-specific proteinase (caspase) family of cysteine proteases, are key enzymes that cause apoptosis; once the caspase is activated, a cascade of caspase ensues, eventually leading to apoptosis. Evidence shows that, as important members of the caspase family, the activation of caspase-3 and caspase-9 is the key to affecting renal cell apoptosis [24, 25] and promotes the extent of damage to kidney cells in response to Ang II, chemotherapy drugs, or oxidative stress [26–28].

Studies have shown that renal injury caused by hypertension is related to the activation of the apoptosis pathway (caspase-3/9) [29]. Kidney tissue damage can be significantly alleviated and prevented by reducing the activity of apoptotic proteins such as cleaved caspase-9 and cleaved caspase-3 [30]. Proteins that promote apoptosis can exacerbate kidney damage caused by inflammation, oxidative stress, or high blood sugar and hypertension [31–33]. This study attempts to explore the mechanism by which miR-205 and miR-206 protect the kidneys from damage caused by hypertension from the perspective that EPC-MVs can carry dysregulated miRNAs to affect disease.

## 2. Methods

### 2.1. Animals

The study was conducted in accordance with the Declaration of Helsinki (as revised in 2013). Six Wistar-Kyoto- and SHR- (male, 10-week-old) specific pathogen-free rats weighing 300-330 g were obtained from the Beijing Vital River Laboratory Animal Technology Co. Ltd (Beijing, China). Wistar-Kyoto rats were used as the control group, and SHR rats as the model group. All rats were placed in a room with 12 h light/dark cycle, the temperature was maintained at 25°C, and the humidity was maintained at 55%; rats have ad libitum access to standard rat food and water in a polystyrene cage. At the end of the experiment, the rats were sacrificed by intraperitoneal injection of sodium pentobarbital (200 mg/kg body weight). Animal experiments were approved by the Animal Care and Use Committee of Hainan Medical University (Haikou, China, approval no. HYLL-2021-053) and were conducted according to the National Institutes of Health guidelines.

### 2.2. Biochemical Analysis of Blood and Urine

On the day before blood sample collection, urine samples were continuously collected from each animal for 24 hours. Blood urea nitrogen (BUN), serum creatinine (Scr), and UAE were measured by standard biochemical kits (BHKT Clinical Reagent Co., Ltd., Beijing, China). Creatinine clearance (CCr) was calculated according to the following formula: CCr = urinary creatinine (mg/ml) × urine output (ml/kg)/plasma creatinine (mg/ml) [34].

### 2.3. Gene Expression Omnibus (GEO) Analysis

The dataset GSE110231 was downloaded and analyzed from the GEO website (https://www.ncbi.nlm.nih.gov/geo/), which included 3 healthy SD rats (GSM2983040, GSM2983041, and GSM2983042) and 3 diabetic rats (GSM2983037, GSM2983038, and GSM2983039). Use Gene Expression Profiling Interactive Analysis 2 (http://gepia2.cancer-pku.cn/#index) to analyze the differential expression of miRNA, and the criteria for differential expression are as follows: *p* < 0.05 and log_2_|*fold* *change*| ≥ 2.

### 2.4. Real-Time Quantitative Polymerase Chain Reaction (RT-qPCR)

According to the manufacturer's instructions, PRKs, EPCs, and MVs were extracted using Trizol reagent (Invitrogen, Thermo Fisher Scientific, Inc.). After 10 minutes of centrifugation (13,000 × g, 4°C) using JIDI-17RS refrigerated centrifuge (Guangzhou JIDI Instrument Co., Ltd, Guangzhou, China), RNA was reverse-transcribed with PrimeScript RT kit (Takara Bio, Japan). SYBR® Premix Ex TaqTM II kit (Takara Bio) was used for RT-qPCR analysis using the Applied Biosystems®7500 Real-Time PCR system (CA, USA). PCR experiments were carried out under the following conditions: 50°C for 25 min, 94°C for 2 min, followed by 25 cycles of 94°C for 30 s, 60°C for 30 s, and 72°C for 2 min. The primer sequences of miR-205, miR-206, and DDX5 used for RT-qPCR are shown in Table 1. Target RNA levels were normalized to those of the housekeeping genes U6 or *β*-actin, and relative levels of miR-205, miR-206, and DDX5 expression were determined using the 2^−*ΔΔ*Ct^ method [35].

### 2.5. Culture and Identification of EPCs

After dissociation of rat femur and tibia, the mixture was rinsed with sterile PBS and collected. Following centrifugation at 4°C (1000 × g, 5 min), EPCs were isolated by Ficoll (Sigma-Aldrich) density gradient centrifugation (1000 × g) and incubated at 37°C and 5% CO_2_ with Endothelial Cell Growth Basal Medium-2 (EGM-2; Lonza, Basel, Switzerland), and the medium was changed after 24 hours. After 4 days, the medium was changed and the adherent cells were cultured for another 3 days [36]. Dil-Ac-LDL (SolarBio, Beijing, China) and PI (Solarbio) costaining were used to identify EPCs.

### 2.6. Preparation and Characterization of EPC-MVs

EPCs were cultured for 7 days as before, washed twice with PBS, and then serum starved for 12 h. Subsequently, EGM-2 medium containing cultured EPCs was centrifuged at 4°C (1000 × g, 15 min), and the supernatant was extracted at 4°C (100 000 × g, 60 min) for the collection of secreted EPC microvesicles. Transmission electron microscopy (TEM) was used to observe whether the collected MVs had double-membrane vesicle-like bodies. After centrifuging the collected medium supernatant at 4°C (1000 × g, 15 min), take the supernatant and extract the supernatant at 4°C (100 000 × g, 60 min) to collect MVs. Extraction of MVs was verified using transmission electron microscopy (TEM). In the coculture system, 50 *μ*g/ml EPC-MVs were added to the top chamber of a transwell assay plate, and PRKs were added to the bottom chamber and incubated for 24 h [37].

### 2.7. Isolation and Culture of PRKs

As shown in our previous study, PRKs were successfully isolated and cultured [37]. Briefly, rat kidney tissue was isolated, a portion was used for RT-qPCR analysis, and a portion of the renal cortex was cut into tissue fragments of approximately ~1 mm^3^. Tissue fragments were digested with collagenase type I (Gibco; Thermo Fisher Scientific, Inc.), and cells were isolated by Ficoll (Sigma-Aldrich; Merck KGaA) density gradient centrifugation, seeded in 6-well plates, and incubated at 37°C and 5% CO_2_. After 24 h, the medium was removed and replaced with fresh medium. Immunofluorescence (IF) identification of PRKs was conducted. In short, PRKs were incubated for 3 passages with *α*-smooth muscle actin (*α*-SMA; 1 *μ*g/ml; cat. no. ab7817; Abcam, Cambridge, England) and vimentin (1 : 250; cat. no. ab92547; Abcam) at 4°C overnight, then treated with Alexa Fluor® 488- (1 : 100; cat. no. ab150077; Abcam) or 647-labeled (1 : 200; cat. no. ab150075; Abcam) secondary antibodies for 1 hour at 37°C. Finally, cells were stained with DAPI (0.5 *μ*g/ml; Solarbio) for 5 min at 25°C. Finally, images were captured using a fluorescence microscope (magnification, ×400; Leica Microsystems GmbH). PRK injury model was constructed by treating with Ang II (1 *μ*M; Sigma-Aldrich) at 37°C for 24 h.

### 2.8. Cell Transfection

Transfect miR-205, miR-206 agomiR/antagomiR, overexpression (ov) DDX5 plasmid, and their negative control (NC) into PRKs or EPCs with Lipofectamine 3000 (Invitrogen) according to the manufacturer's instructions, and incubate in the dark for 4 hours (37°C, 5% CO_2_). After changing the medium for 24 or 48 hours, collect the cells or supernatant for further processing. All RNAs were purchased from Sangon Biotech Co., Ltd (Shanghai, China), and their sequences are shown in Table 2.

### 2.9. EPC-MVs and PRK Fusion

EPC-MVs (50 g/ml) were combined with 2 ml PKH26 (2 × 10^−6^ M; Sigma-Aldrich) and incubated at 25°C for 5 min. After centrifugation (4°C, 120,000 × g, 60 min), the pellet was resuspended in EGM-2 medium, then added to PRK, and incubated at 37°C for 24 hours. Finally, add 1 *μ*g/ml DAPI for nuclear staining at 25°C for 15 minutes. Cell images were acquired using a fluorescence microscope (magnification, ×400).

### 2.10. Cell Proliferation Assays

According to the manufacturer's instructions, 10 *μ*l of Cell Counting Kit-8 reagent (CCK-8, Solarbio) was added to PRKs at 0 h and 24 h, respectively, and incubated for 1 h. The optical density at 490 nm was then measured with a microplate reader (Multiskan MK3, Thermo Fisher Scientific, Inc.) to assess the cell proliferation rate.

### 2.11. Western Blotting

After lysis of PRKs or MV with radio immunoprecipitation assay lysis buffer, the lysate protein concentration was determined using a bicinchoninic acid protein detection kit (Solarbio) and resolved using 10% SDS-polyacrylamide gel electrophoresis (Solarbio). Protein bands were transferred onto a polyvinylidene fluoride membrane, blocked with 5% BSA (Solarbio), and incubated with CD63 (1 : 1000; ab134045, Abcam), CD81 (1 : 1000; ab109201, Abcam), Tsg101 (1 : 1000; ab125011, Abcam), DDX5 (1 : 1000; ab128928, Abcam), cleaved caspase-3 (1 : 500; ab32042, Abcam), and cleaved caspase-9 (1 : 1000; 9505T, Cell Signaling Technology Co., LTD; Danvers, Massachusetts, USA) primary antibodies overnight (4°C). Then, they were rinsed with TBST buffer (Solarbio, contains 0.05% Tween-20) twice, for 10 min each time, and incubated with the secondary antibody for 90 min at 25°C. Subsequently, use ECL (enhanced chemiluminescence) reagent for chemiluminescence reaction. Finally, they were developed and fixed using Developer and Fixer Kit (Beyotime, Shanghai, China).

### 2.12. Dual Luciferase

The miRDB database (http://mirdb.org/) was used to predict binding sites between miR-205 or miR-206 and DDX5. PRKs were transfected with miR-205 or miR-206 and their negative controls, vector plasmid containing wild-type (WT) or mutant (mut) DDX5, and pRL-SV40 reporter vector plasmid. Transfected cells were incubated for 48 h, and luciferase activity was measured at 490 nm according to dual-luciferase reporter assay system (Promega, USA) instructions.

### 2.13. Detection of Cell Apoptosis Using Flow Cytometry (FCM)

A dose of 5 *μ*l of Annexin V-Fluorescein Isothiocyanate (FITC; BD Biosciences, CA, USA) and 10 *μ*l of Propidium Iodide (PI; BD Biosciences) was incubated with PRKs (0.4 × 10^5^ cells/ml) in the dark (25°C, 15 min), then rinsed twice with PBS (Gibco; Thermo Fisher Scientific, Inc.).

### 2.14. Statistical Analyses

All experiments were repeated three times. Data are expressed as the mean ± standard deviation (SD). Differences between multiple groups were assessed using one-way analysis of variance and Bonferroni post hoc test. Student's *t*-test was used for independent two-group analyses. Statistical significance was set at *p* < 0.05.

## 3. Results

### 3.1. Screening of Potential Therapeutic miRNAs

**Figure 1:**
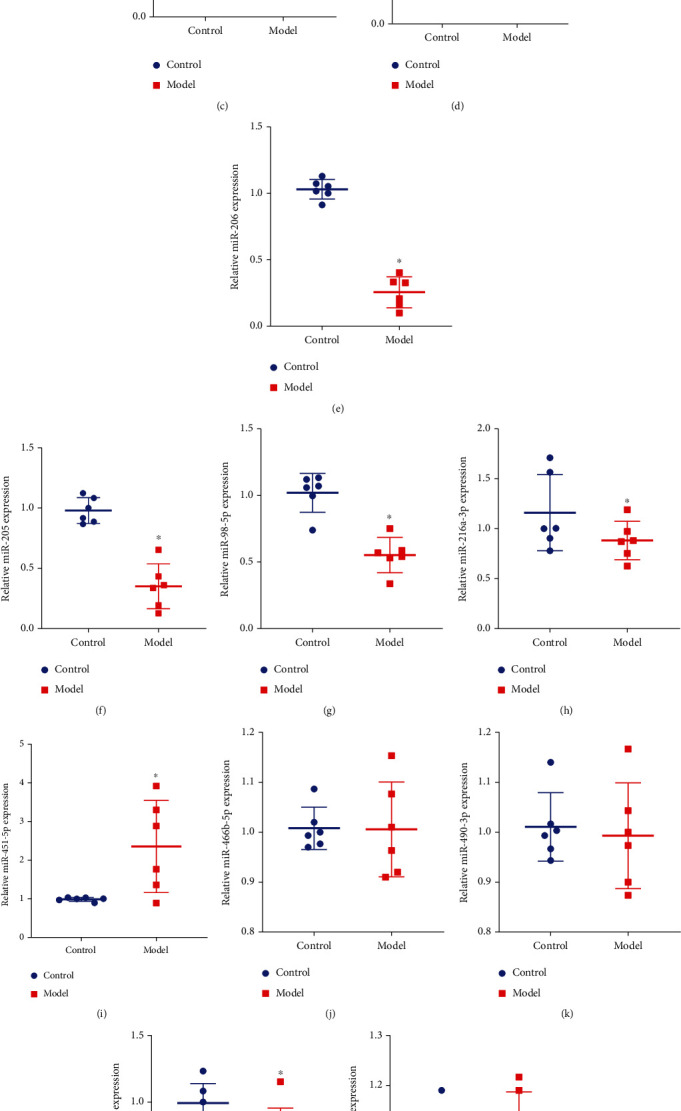
Screening of potential therapeutic miRNAs. (a) Determination of UAE in rats. (b) The sample GSE110231 was analyzed by GEO to screen miRNAs. Heat map shows differentially expressed miRNAs in diabetic rats compared with healthy rats. The blue band represents low expression, and the red band represents high expression. The blue part is low expression, and the red part is high expression. (c–o) RT-qPCR analysis of miR-1-3p (c), miR-9a-5p (d), miR-206 (e), miR-205 (f), miR-98-5p (g), miR-216a-3p (h), miR-451-5p (i), miR-466b-5p (j), miR-490-3p (k), miR-743b-3p (l), miR-881-3p (m), miR-1949 (n), and miR-219a-2-3p (o) expression in vivo. ^∗^*p* < 0.05. GEO: Gene Expression Omnibus; RT-qPCR: real-time quantitative polymerase chain reaction; UAE: urinary albumin excretion; miR: microRNA.

After continuous feeding of SHR rats for 4 weeks, the UAE of SHR rats was significantly higher than that of normal rats, and the UAE value was 39.27 ± 9.07 mg/24 h, which reached the standard of HN model (Figure 1(a)). Through GEO analysis of the GSE110231 dataset, it was found that there were 13 differentially expressed rat-derived miRNAs (Figure 1(b)), namely, miR-1-3p, miR-9a-5p, miR-98-5p, miR-205, miR-206, miR-216a-3p, miR-451-5p, miR-466b-5p, miR-490-3p, miR-743b-3p, miR-881-3p, miR-1949, and miR-219a-2-3p. The kidney tissues of normal rats and rats with HN were taken, and RT-qPCR analysis of these 13 miRNAs was performed, respectively. The results showed that compared with control, miR-1-3p, miR-9a-5p, miR-98-5p, miR-205, miR-206, miR-216a-3p, miR-219a-2-3p, miR-743b-3p downregulated, miR-451-5p, miR-1949 upregulated, miR-219a-2-3p, miR-490-3p, miR-466b-5p, miR-881-3p, and miR-219a-2-3p had no difference (Figures 1(c)–1(o)). We selected miR-205 and miR-206 as potential therapeutic targets and synthesized miR-205 and miR-206 agomiR/antagomiR.

### 3.2. EPC Isolation and Identification

**Figure 2:**
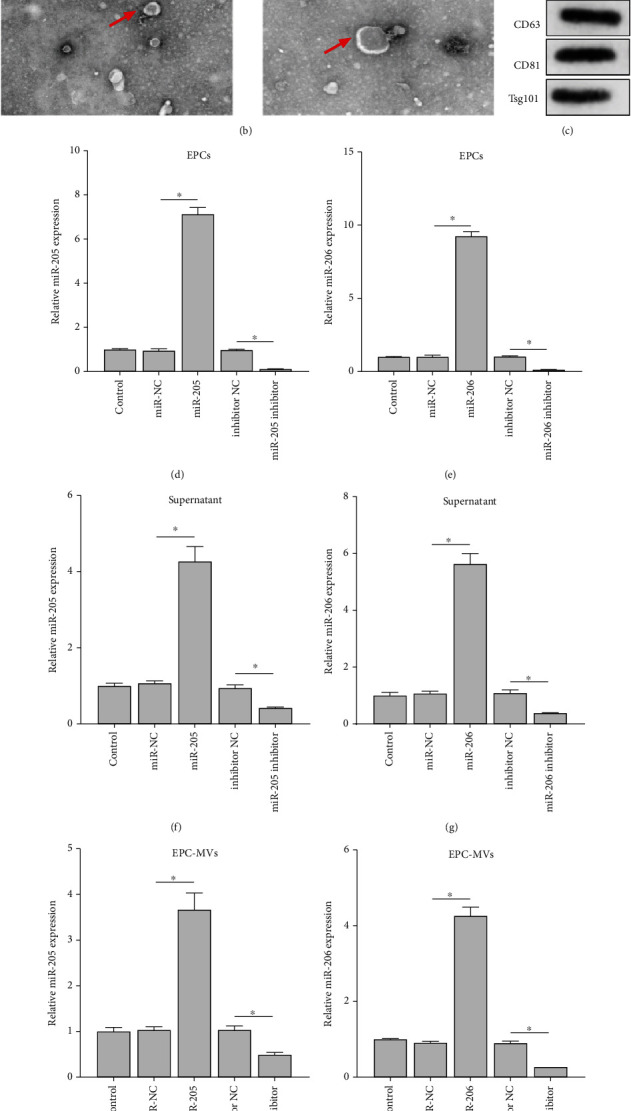
EPCs carry dysregulated miR-205 and miR-206 through the secreted MV pathway. (a) Dil-Ac-LDL staining was used to analyze the separation of EPCs. Blue is DAPI staining, and red is Dil-Ac-LDL staining. (b) TEM analysis of the extraction of MVs. Magnification: 40,000x. (c) Western blot analysis of the protein expression of CD63, CD81, and Tsg101 in the extracted MVs. (d–i) RT-qPCR analysis of the expression changes of miR-205 and miR-206 in EPCs (d, e), supernatants (f, g), and MVs (h, i). ^∗^*p* < 0.05. Dil-Ac-LDL: Dil complex acetylated low-density lipoprotein; DAPI: 4′,6-diamidino-2-phenylindole; EPCs: endothelial progenitor cells; MVs: microvesicles; RT-qPCR: real-time quantitative polymerase chain reaction; TEM: transmission electron microscopy; IF: immunofluorescence; miR: microRNA.

Isolated EPCs were identified using Dil-Ac-LDL staining. The red fluorescence (Dil-Ac-LDL) coincided with blue fluorescence (DAPI nuclear staining) by more than 90% (Figure 2(a)), confirming the successful separation of EPCs. After the medium was collected, MVs were extracted and observed under TEM. TEM results observed double-membrane vesicle-like bodies (Figure 2(b)). After western blot analysis, the protein levels of CD63, CD81, and Tsg101 were all positive (Figure 2(c)), so we determined that MVs were successfully extracted. miR-205 and miR-206 construct agomiR/antagomiR transfected EPC and collected supernatant and secreted MVs. The results showed that miR-205 and miR-206 were upregulated in the agomiR-transfected group and downregulated in the antagomiR-transfected group regardless of EPCs, supernatant, or MVs (Figures 2(d)–2(i)). Therefore, we confirmed that the synthetic miR-205 and miR-206 agomiR/antagomiR were effective, and the expression regulation of both was transmitted to MVs through EPCs.

### 3.3. Therapeutic Effects of miR-205 or miR-206-MVs on the Kidney In Vivo

**Figure 3:**
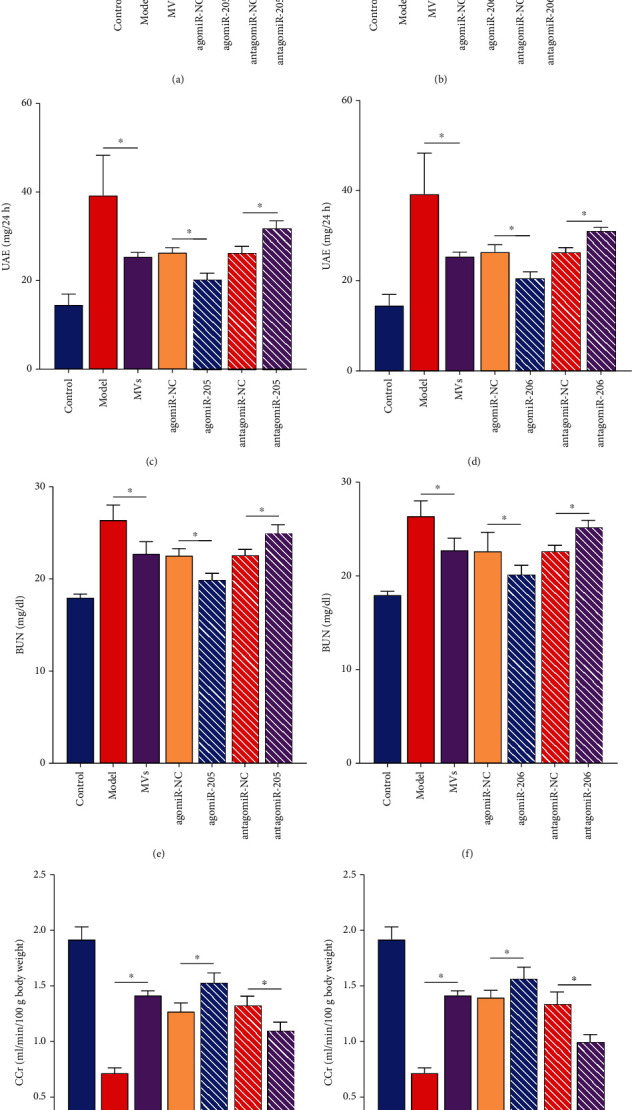
Kidney protection of miR-205 and miR-206-MVs in vivo. (a, b) RT-qPCR validated the effects of dysregulated miR-205 (a) and miR-206 (b) MVs on the expression of miR-205 and miR-206 in vivo. (c–h) Standard biochemical kits were used to analyze the effects of miR-205 and miR-206-MVs on UAE (c, d), BUN (e, f), and CCr (g, h) in vivo. ^∗^*p* < 0.05. RT-qPCR: real-time quantitative polymerase chain reaction; miR: microRNA; UAE: urinary albumin excretion; BUN: blood urea nitrogen; CCr: creatinine clearance.

The collected MVs were injected intravenously into rats. We observed that in rats injected with miR-205 and miR-206 MVs, the expressions of miR-205 and miR-206 were upregulated in the agomiR-injected group and downregulated in the antagomiR-injected group compared to the MV group (Figures 3(a) and 3(b)). UAE and BUN indicators were upregulated, and CCr decreased in HN. EPC-MVs significantly repaired the damage caused by HN, and this repair ability was amplified by miR-205 or miR-206 and was reduced by MVs that inhibited the expression of miR-205 or miR-206 (Figures 3(c)–3(h)). Therefore, we can confirm that miR-205 and miR-206 are therapeutic targets for HN, and EPCs regulate renal injury caused by HN in vivo by secreting MVs expressing miR-205 and miR-206. The molecular mechanism of action is still unclear.

### 3.4. MVs Protect PRKs from Ang II-Induced Damage

**Figure 4:**
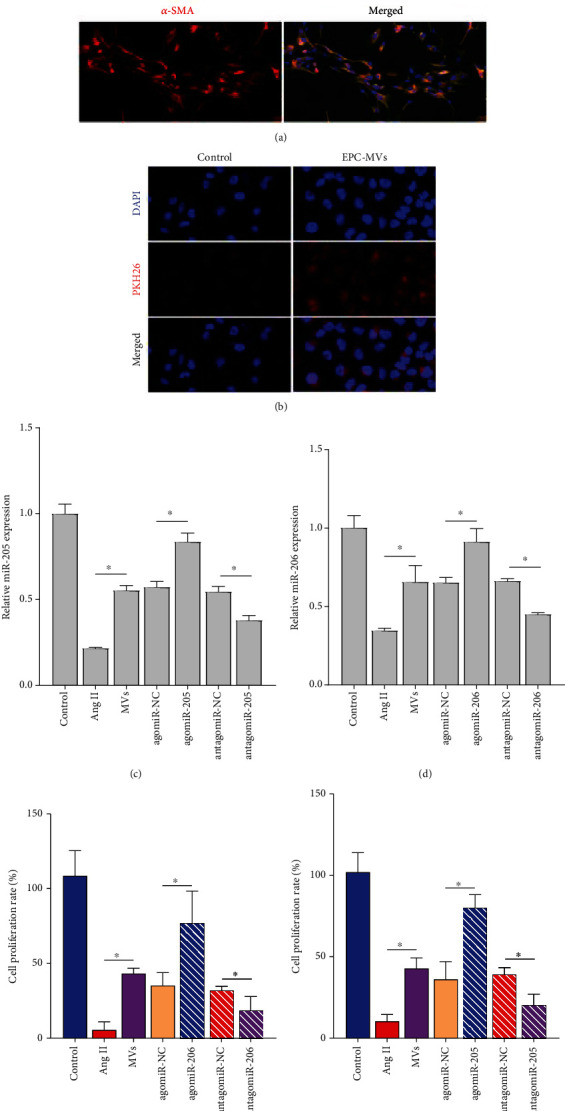
miR-205 and miR-206-MVs protect PRKs from Ang II damage in vivo. (a) IF analysis of the expression intensity of vimentin and *α*-SMA in the isolated PRKs. Blue is DAPI, green is anti-vimentin antibody, and red is anti-*α*-SMA antibody. (b) Fluorescence microscopy was used to observe the fusion of PKH26-labeled EPC-MVs and PRKs. Blue is DAPI, and red is MVs carrying PKH26-labeled. (c, d) RT-qPCR analysis in vitro miR-205 and miR-206-MVs regulate the expression of miR-205 and miR-206 in PRKs. (e, f) CCK8 analysis of the effects of miR-205 and miR-206-MVs on the proliferation of PRKs induced by Ang II in vitro. ^∗^*p* < 0.05. RT-qPCR: real-time quantitative polymerase chain reaction; miR: microRNA; CCK-8: Cell Counting Kit-8; Ang II: angiotensin II; EPCs: endothelial progenitor cells; PRKs: primary rat kidney cells; MVs: microvesicles; IF: immunofluorescence; *α*-SMA: *α*-smooth muscle actin.

PRKs from normal rats were isolated, and the isolated PRKs were analyzed using IF staining (vimentin/*α*-SMA). The results showed that vimentin and *α*-SMA were highly expressed in PRKs, indicating that the isolation of PRKs was successful (Figure 4(a)). When MVs labeled with PKH26 were added to PRKs, we observed that red fluorescence of PKH26 was detected in the cytoplasm of PRKs, confirming that MVs could be incorporated into PRKs (Figure 4(b)). Ang II induction was added to mimic an in vitro HN model, and MVs carrying dysregulated miR-205 and miR-206 were fused to PRKs. Adding Ang II induction to mimic the HN model in vitro, Ang II mediated the expression inhibition of miR-205 and miR-206, and EPC-MVs reduced the effect of Ang II. The effect of EPC-MVs was enhanced by miR-205 and miR-206 agomiR and attenuated by miR-205 and miR-206 antagomiR (Figures 4(c) and 4(d)). Growth inhibition promoted by Ang II was suppressed by normal MVs. The action of normal MVs was promoted by MVs carrying miR-205 and miR-206 agomiR and suppressed by MVs carrying miR-205 and miR-206 antagomiR (Figures 4(e) and 4(f)).

### 3.5. miR-205 and miR-206 Were Negatively Correlated with DDX5

**Figure 5:**
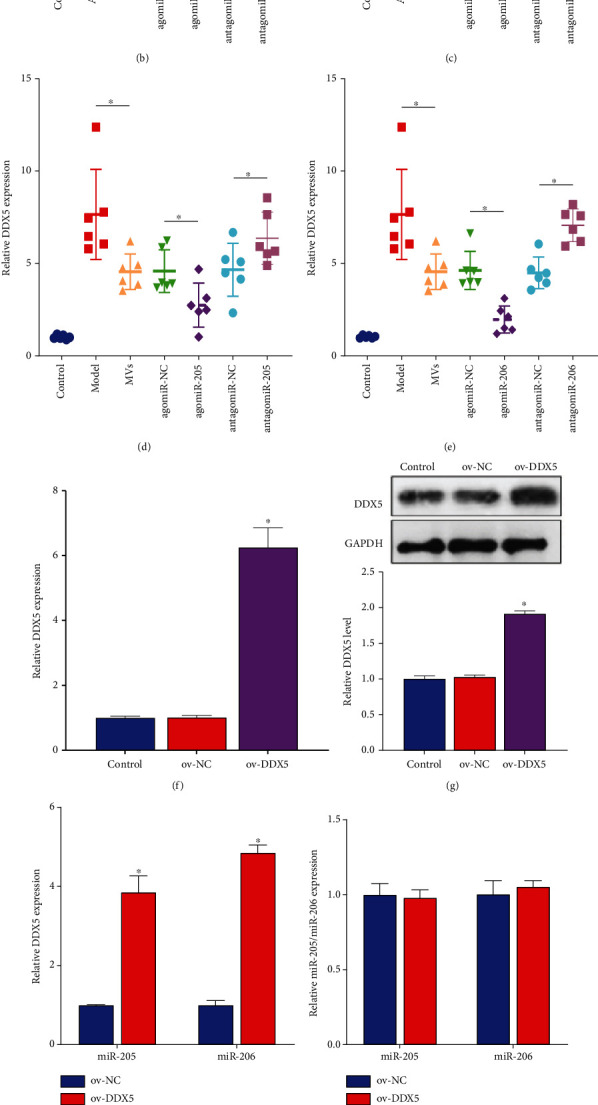
The mechanism by which miR-205 and miR-206 work is achieved by targeting downstream DDX5. (a) The miRDB database analyzed the common downstream targets of miR-205 and miR-206. (b, c) RT-qPCR analysis in vitro miR-205 (b) and miR-206 (c) MVs regulate DDX5 expression. (d, e) RT-qPCR analysis in vivo miR-205 (d) and miR-206 (e) MVs regulate DDX5 expression. (f) RT-qPCR verified the validity of the overexpressed DDX5 plasmid. (g) Western blot verified the effectiveness of overexpressing DDX5 plasmid. (h) RT-qPCR analysis of the effect of overexpressing DDX5 in the presence of miR-205 or miR-206 agomiR on DDX5 expression. (i) RT-qPCR analysis of the effect of overexpressing DDX5 in the presence of miR-205 or miR-206 agomiR on miR-205 or miR-206 expression. (j, k) The miRDB database analysis of the binding sites of miR-205 (j) or miR-206 (k) to DDX5. (l, m) Dual-luciferase assays for direct binding of miR-205 (l) or miR-206 (m) to DDX5. ^∗^*p* < 0.05. RT-qPCR; real-time quantitative polymerase chain reaction; miR: microRNA; ov: overexpression; DDX5: DEAD-box helicase 5; EPCs: endothelial progenitor cells; PRKs: primary rat kidney cells; MVs: microvesicles; WT: wild type; mut: mutant.

The miRDB database (http://mirdb.org/) screened putative targets and found that miR-205 and miR-206 share a common target, DDX5 (Figure 5(a)). Subsequent RT-qPCR results showed that Ang II induced upregulation of DDX5 expression in PRKs, and the addition of EPC-MVs suppressed the effect of Ang II. The effect of EPC-MVs was enhanced by miR-205 and miR-206 agomiR and attenuated by miR-205 and miR-206 antagomiR (Figures 5(b) and 5(c)). Similar to the in vitro results, the expression of DDX5 was downregulated in the agomiR-transfected group and upregulated in the antagomiR group (Figures 5(d) and 5(e)). Therefore, miR-205 and miR-206 were negatively correlated with the expression of DDX5. We then constructed plasmids overexpressing DDX5 and transfected them into PRKs. The results showed that compared with the control group ov-NC, the gene and protein levels of overexpressing DDX5 were significantly upregulated (Figures 5(f) and 5(g)), so the construction of the plasmid overexpressing DDX5 was successful. We transfected ov-DDX5 in miR-205 and miR-206 mimic, and the results showed that the presence of miR-205 and miR-206 mimic suppressed the expression of DDX5 compared to ov-NC, but the addition of ov-DDX5 made this effect reversed (Figure 5(h)), while the expression of miR-205 and miR-206 did not change significantly (Figure 5(i)).

### 3.6. miR-205 and miR-206 Directly Target DDX5

Bioinformatics predicted binding sites of DDX5 to miR-205 or miR-206 (Figures 5(j) and 5(k)). The mechanism through which miR-205 and miR-206 modulate DDX5 expression was examined by introducing the 3′-UTR of DDX5 containing a joint binding site of miR-205 and miR-206 downstream of the luciferase reporter. Subsequently, the results of dual-luciferase analysis showed that the luciferase activity of WT DDX5 3′-UTR construct in cells transfected with miR-205 or miR-206 mimic was significantly lower compared with that in cells transfected with the mimic NC. A luciferase reporter containing the mut DDX5 3′-UTR binding site of miR-205 or miR-206 was also constructed. The luciferase activity of the mut DDX5 3′-UTR construct was not suppressed upon transfection with miR-205/miR-206 mimic or inhibitor (Figures 5(l) and 5(m)). These results show that miR-205 and miR-206 cotarget DDX5 and inhibit the transcriptional activity of DDX5 by binding to the 3′-UTR of DDX5.

### 3.7. Effects of the miR-205/miR-206-DDX5 Axis on Proapoptotic Proteins

**Figure 6:**
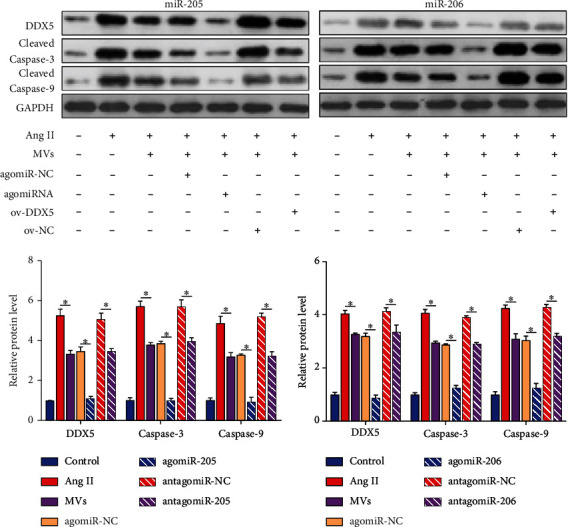
miR-205 and miR-206-MVs regulate the activation of apoptotic proteins. Western blot analysis of overexpression of DDX5 in the presence of miR-205 or miR-206 agomiR on the protein levels of DDX5, caspase-3/9. ^∗^*p* < 0.05. miR: microRNA; ov: overexpression; DDX5: DEAD-box helicase 5; EPCs: endothelial progenitor cells; PRKs: primary rat kidney cells; MVs: microvesicles; Ang II: angiotensin II.

**Figure 7:**
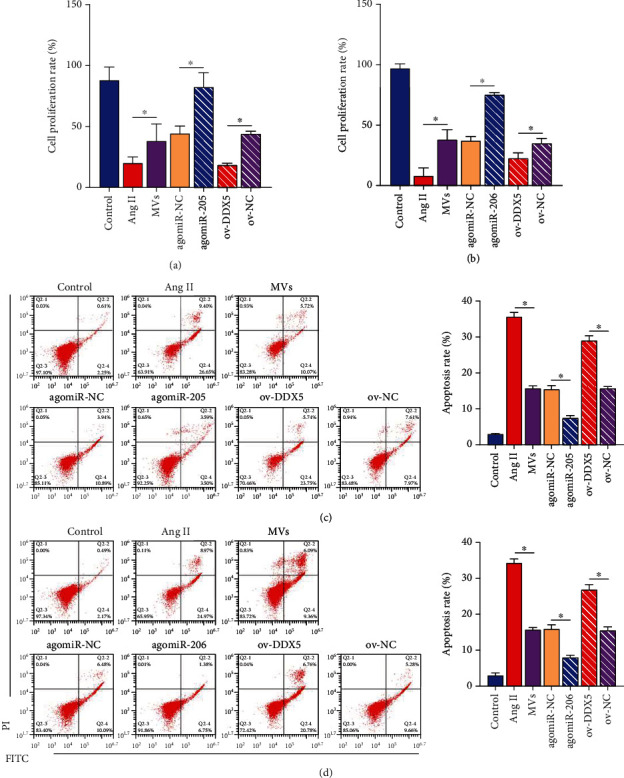
Effects of miR-205 and miR-206 interacting with DDX5 on the proliferation and apoptosis of PRKs. (a, b) CCK8 analysis of the effect of overexpressing DDX5 on the proliferation rate of PRKs in the presence of miR-205 (a) and miR-206 (b) agomiR. (c, d) FCM analysis of the effect of overexpressing DDX5 on the apoptosis of PRKs in the presence of miR-205 (c) and miR-206 (d) agomiR. ^∗^*p* < 0.05. RT-qPCR: real-time quantitative polymerase chain reaction; miR: microRNA; ov: overexpression; DDX5: DEAD-box helicase 5; Ang II: angiotensin II; FCM: flow cytometry; EPCs: endothelial progenitor cells; PRKs: primary rat kidney cells; MVs: microvesicles; CCK-8: Cell Counting Kit-8.

By western blot analysis, we confirmed that Ang II promoted DDX5 protein level and the activation of proapoptotic protein caspase-3/9, and EPC-MVs had a significant inhibitory effect on this effect. The efficacy of EPC-MVs was enhanced in the overexpressed state of miR-205 or miR-206 and was attenuated by overexpression of DDX5 (Figure 6). Furthermore, EPC-MVs inhibited Ang II-mediated PRK growth inhibition and apoptosis promotion. miR-205 or miR-206 enhanced the utility of EPC-MVs, whereas overexpression of DDX5 inhibited the utility of miR-205 or miR-206 MVs (Figures 7(a)–7(d)).

## 4. Discussion

In the present study, we demonstrated for the first time that EPC-MVs carrying high levels of miR-205 and miR-206 could protect kidneys or PRKs from cellular damage caused by hypertension or Ang II.

The proliferation and angiogenesis and anti-inflammatory abilities of EPCs can effectively protect the kidneys of patients with nephropathy [37, 38] and play an important role in maintaining vascular integrity and improving renal function [6]. Recent evidence suggests that EPCs play a role in protecting renal function through secreted MVs [7]. MVs are anti-inflammatory, improve endothelial function, and alleviate endothelial dysfunction caused by oxidative stress [39, 40]. Since the damaged kidneys cannot effectively filter the metabolic wastes in the blood, which eventually leads to the occurrence of kidney disease [41], this paper conducted a GEO analysis on the diabetic rats based on the possibility of kidney disease in the diabetic rats, and the data was used to analyze the same kidney damage caused by hypertension. One of the main conclusions of this study is that MVs secreted by EPCs reduced the rise in UAE and BUN and the decrease in CCr induced by HN in vivo. In vitro, MVs promoted the proliferation of PRKs and inhibited apoptosis; all these evidences confirmed that MVs can improve renal function. This is consistent with the conclusions reached by our previous study [37].

It has been reported that MVs can deliver miRNAs to cells, thereby affecting disease development. For example, MVs carrying miR-148a can regulate adipogenic and osteogenic differentiation by targeting the receptor tyrosine kinase-like orphan receptor 2 pathway [42]. miR-191 secreted by MVs induces renal tubular epithelial cell apoptosis by inhibiting cystathionine-*β*-synthase [43]. This study confirmed that both miR-205 and miR-206 secreted by MVs could promote the improvement of HN by MVs. The secreted low-expressed miR-205 and miR-206 inhibited the effect of MVs. The results in in vitro experiments were also similar; to be specific, MVs secreted miR-205 and miR-206 to promote MVs to ameliorate Ang II-induced PRK growth inhibition and apoptosis, and low expression of miR-205 and miR-206 partially inhibited the effect of MVs. Furthermore, after transfection of miR-205 or miR-206 agomiR/antagomiR, deregulated expression of miR-205 or miR-206 was detected in EPCs, supernatants, and MVs, implying that MVs carried deregulated miR-205 or antagomiR miR-206 is produced by secretion from EPCs.

To further explore the mechanism by which miR-205 or miR-206 exert their effects, we analyzed their common downstream target DDX5 by bioinformatics. Studies have confirmed that miR-206 improves renal function by binding to DDX5 [23]. Here, we report that miR-205 and miR-206 together target DDX5. The growth promotion and inhibition of PRKs by miR-205 and miR-206 could be reversed by overexpressed DDX5, and the restriction of transcriptional activity and translational level of DDX5 by miR-205 and miR-206 was also alleviated by overexpressed DDX5. Therefore, DDX5 is negatively regulated with miR-205 and miR-206.

Increasing evidence suggests that abnormal activation of apoptotic proteins may be a major factor in causing kidney damage [31, 44]. Our study shows that MVs can reduce the activation of apoptotic protein caspase-3/9. Secretion of miR-205 and miR-206 enhanced the effect of MVs, while overexpression of DDX5 inhibited the enhancement of MVs by miR-205 and miR-206.

This study has some limitations. First, there are no clinical data on the treatment of EPC-MVs in HN. Second, changes in various signaling pathways, especially those related to inflammation or oxidative stress, have not been further explored. All of these will be the focus of our future research.

In conclusion, EPCs protect against hypertension-induced renal injury by secreting MVs carrying dysregulated miR-205 and miR-206 expression. The mechanism is related to miR-205 and miR-206 cotargeting DDX5 and promoting cell growth inhibition and apoptosis. This provides a new therapeutic strategy for HN.

## Figures and Tables

**Table 1:** Sequences of RT-qPCR primer.

Gene symbol	5′--3′
miR-1-3p forward	ACACTCCAGCTGGGTGGAATGTAAAGAAGT
miR-9a-5p forward	ACACTCCAGCTGGGTCTTTGGTTATCTAGCT
miR-98-5p forward	ACACTCCAGCTGGGTGAGGTAGTAAGTTGT
miR-205 forward	ACACTCCAGCTGGGTCCTTCATTCCACCGGA
miR-206 forward	ACACTCCAGCTGGGTGGAATGTAAGGAAGT
miR-216a-3p forward	ACACTCCAGCTGGGCACAGTGGTCTCTGGG
miR-451-5p forward	ACACTCCAGCTGGGAAACCGTTACCATTAC
miR-466b-5p forward	ACACTCCAGCTGGGTATGTGTGTGTGTATG
miR-490-3p forward	ACACTCCAGCTGGGCAACCTGGAGGACTCC
miR-743b-3p forward	ACACTCCAGCTGGGGAAAGACACCATACTG
miR-881-3p forward	ACACTCCAGCTGGGTAACTGTGGCATTTCT
miR-1949 forward	ACACTCCAGCTGGGTATACCAGGATGTCAGC
miR-219a-2-3p forward	ACACTCCAGCTGGGAGAATTGTGGCTGGAC
Universal reverse primer for miRNA	CTCAACTGGTGTCGTGGA
U6 forward	CTCGCTTCGGCAGCACA
U6 reverse	AACGCTTCACGAATTTGCGT
DDX5 forward	CGACCAAAACCCGTCAAAGG
DDX5 reverse	CCGAAGCTGCACTACGGAA
*β*-Actin forward	CACCCGCGAGTACAACCTTC
*β*-Actin reverse	CCCATACCCACCATCACACC

DDX5: DEAD-box helicase 5; RT-qPCR: real-time quantitative polymerase chain reaction; miR: microRNA.

**Table 2:** A list of miRNA's sequences.

Gene symbol	Sequences
agomiR-NC	UGCAUCACUCGUUGCGUCCUAUC
antagomiR-NC	GACTTAAGGAAGGTAGCCGGAAC
agomiR-205	UCCUUCAUUCCACCGGAGUCUGU
antagomiR-205	ACAGACTCCGGTGGAATGAAGGA
agomiR-206	UGGAAUGUAAGGAAGUGUGUGG
antagomiR-206	CCACACACTTCCTTACATTCCA

RT-qPCR: real-time quantitative polymerase chain reaction; miR: microRNA; NC: negative control.

## Data Availability

The data used to support the findings of this study are included within the article.
